# Continuing Professional Development – Medical Imaging

**DOI:** 10.1002/jmrs.70037

**Published:** 2025-11-21

Maximise your continuing professional development (CPD) by reading the following selected article and answering the five questions. Please remember to self‐claim your CPD and retain your supporting evidence. Answers will be available via the QR code and published in JMRS—Volume 73, Issue 4, December 2026.

## Enhancing Patient‐Centred Care and Cultural Safety in Medical Imaging: The Radiographers' Experience of Communicating With Patients in a Multicultural and Multilingual Setting in Auckland

Nica Abrasado, Sibusiso Mdletshe, https://doi.org/10.1002/jmrs.70005.

1. Culturally safe practice is a crucial component of medical imaging and radiation therapy. Which of the following best represents a key aspect of culturally safe practice?
  - Patients acknowledging radiographers as trustworthy professionals.
  - Professionals engaging in self‐reflection on their own culture, attitudes, and beliefs about others.
  - Ensuring that patients follow instructions without question.
  - Focusing solely on equality for all patients.
2. Which of the following is an effective approach to enhancing patient‐centred care and promoting positive health outcomes, particularly within Indigenous communities or ethnic minority groups?
  - Involving family members to provide additional support and improve communication during imaging or treatment procedures.
  - Excluding family members from imaging or treatment procedures to prioritise patient privacy.
  - Communicating with patients using formal English to ensure better understanding.
  - Only allowing patients to be accompanied by their nurse or healthcare professional during procedures.
3. Cultural competence in healthcare goes beyond following protocols. Which of the following most accurately defines its core purpose?
  - Ensuring patients follow the care plan, provided it aligns with their cultural and religious beliefs.
  - Verifying the accuracy of the patient's medical history with their confirmation.
  - Encouraging patients to learn your language during interactions in imaging or treatment.
  - The ability to understand, communicate, and interact effectively with individuals from diverse backgrounds, while respecting their beliefs, cultures, and practices.
4. Which of the following approaches is most likely to improve communication with patients from diverse cultural backgrounds?
  - Teaching patients the language commonly used within the department.
  - Applying adaptive communication strategies to meet individual needs.
  - Avoiding the use of ad‐hoc interpreters due to concerns about reliability.
  - Maintaining eye contact with the patient during conversations.
5. Why is it important to consult with local Indigenous or ethnic minority stakeholders when conducting research?
  - To ensure potential participants are more likely to agree to take part in the study.
  - Because it is an ethical requirement and promotes local responsiveness in the research process.
  - To gain access to information that may otherwise be difficult for the research team to obtain.
  - So that stakeholders can receive donations from the research team to support their communities.

## Answers

Scan this QR code to find the answers.

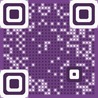
